# Genome-Wide Analysis on the Landscape of Transcriptomes and Their Relationship With DNA Methylomes in the Hypothalamus Reveals Genes Related to Sexual Precocity in Jining Gray Goats

**DOI:** 10.3389/fendo.2018.00501

**Published:** 2018-08-30

**Authors:** Feng Su, Xiaoli Guo, Yanchao Wang, Yuding Wang, Guiling Cao, Yunliang Jiang

**Affiliations:** ^1^Shandong Provincial Key Laboratory of Animal Biotechnology and Disease Control and Prevention, College of Animal Science and Veterinary Medicine, Shandong Agricultural University, Taian, China; ^2^College of Agronomy, Liaocheng University, Liaocheng, China

**Keywords:** Jining Gray goats, sexual precocity, transcriptome, DNA methylome, integrated analysis

## Abstract

The Jining Gray goat is famous for its sexual precocity; however, the exact regulatory mechanism is still unknown. The hypothalamus is the key centrum in the process of animal reproduction, especially in signal transduction, and the initiation of puberty. The identification of potential genes and pathways in the hypothalamus of Jining Gray goat is critical to understanding the regulatory mechanism of sexual precocity in these goats. In this study, mRNA transcriptome analysis of the hypothalamus of juvenile and pubertal goats revealed eight genes (*NTS, ADORA1, CRH, UCN3, E2F2, PDGFRB, GNRH1*, and *CACNA1C*) and three pathways [neuroactive ligand-receptor interaction; gonadotropin-releasing hormone (GnRH) signal; melanoma] that are involved in this regulation. Subsequent methylation analysis on differentially methylated region (DMR) genes revealed the potential regulation network that influences pubertal onset. Correlation analysis verified the methylation level of some DMR genes correlates negatively with expression level. Integrated analysis between transcriptomes and methylomes identified 80 candidate genes involved in GnRH and neuroactive ligand signal pathways, of which *CACNA1C* and *CRH* were differentially expressed genes (DEGs) influenced by methylation level. The *GnRH* gene was the only DEG not affected by its methylation level. In summary, in this study, we identified eight genes and three pathways that are related to pubertal onset in Jining Gray goats, and the expression of *CACNA1C* and *CRH* genes of the GnRH and neuroactive ligand signal pathways were influenced by DNA methylation, while that of the *GnRH* gene was not affected.

## Introduction

The Jining Gray goat, one of the local breeds in the southwest of Shandong province, China, is characterized by its fertility performance. Different from other breeds, the Jining Gray goat is famous for its sexual precocity, annual estrus and multiple gestation features (1). Jining Gray goats reach puberty (time to first ovulation) at the age of 2 months, which is much earlier than for Hu sheep (6 months of age). Moreover, the sexual maturity time of Jining Gray goats (90 days) is significantly earlier than for Boer goats (157.2–191.1 days) (2), Angora goats (180–240 days) and Inner Mongolia Cashmere goats (3). This performance has been extensively applied to increase goat husbandry production; however, the underlying biological mechanisms remain unknown.

The hypothalamus-pituitary-gonadal (HPG) axis plays a critical role in the onset of puberty. As a critical central nervous system in primates, the hypothalamus has key effects in the process of animal reproduction. Secretion capacity is an important feature of the hypothalamus, in which neurosecretory neurons can pulsatile secrete gonadotropin-releasing hormone (GnRH) that regulates follicle-stimulating hormone (FSH) and luteinizing hormone (LH) secretion in the pituitary gland, and is recognized as a signal of puberty onset (4, 5). GnRH secretion is markedly suppressed prior to puberty, whereas at the beginning of puberty, the hypothalamic gonadostat is also depressed and the amplitude of the GnRH pulses increases (5). Existence and release of gonadotropins maintains the mammalian estrous cycle, and influences oocyte development and ovulation in female mammals (6). Another critical function of the hypothalamus is related to its complicated regulatory mechanism, the major regulation of FSH and LH secretion through stimulating hypothalamic GnRH and the feedback from the gonadal gland (7, 8), and the putative LH regulator PACAP regulation pathway that contribute to the oocyte maturation and ovulation (9, 10). Additionally, a complex network of genes is responsible for the control of puberty, such as *KISS1, MKRN, TAC3* (tachykinin3), and *GPR54* (G-protein-coupled receptor 54) genes, whose epigenetic regulation has emerged as having an important role in the regulation of puberty onset. However, the physiological role of the hypothalamus in the accurate regulation of gonadotropin secretion is still poorly understood.

Conventionally, DNA microarray technology is used to identify differentially expressed genes (DEGs) between groups of individuals belonging to contrasting classes of a phenotypic trait of interest (7). High-throughput sequencing technologies have delivered a step change in our ability to hunt for DEGs (8). Traditionally, fold changes were the only criteria reflecting the DEGs and the important regulatory factors were frequently neglected. Gene expression is regulated by a complex interplay between transcription factors, chromatin remodeling processes, and epigenetic modifications of DNA and histones, the core components of chromatin (9). DNA methylation, the addition of a methyl group to a cytosine that predominantly occurs in transposons and other repetitive DNA elements, primarily serves as an epigenetic silencing mechanism (10). It serves as a regulator in the proper regulation of gene expression and gene silencing in normal cells and often participates in long-term developmental gene silencing. Profiling DNA methylation across the genome is important to understand the role of DNA methylation changes during developmental phenotypes (11). Thus, integrated analysis of the relationship of DNA methylation and gene expression in the hypothalamus is necessary to explain the excellent fertility performance of Jining Gray goats.

In this study, we report what is believed to be the first comprehensive and integrative analysis of the transcriptome and genome-wide promoter DNA methylation that underpin the sexual precocity phenotype of Jining Gray goats. We also provide the transcriptome, promoter DNA methylome and locus-specific changes upon the key genes of Jining Gray goat hypothalamus. Moreover, using these data, we have unveiled several candidate genes and signal pathways related to sexual precocity in Jining Gray goats.

## Materials and methods

### Ethics statement

All goats in this study were housed in open sheepfolds and fed *ad libitum*. The sacrifice of goats used sodium barbital after anesthesia. All procedures involving animals were approved by the Animal Care and Use Committee of Shandong Agricultural University.

### Sample collection and preparation

All the goats in this study were housed in open sheepfolds. The juvenile goats were 1 month old, weighing 4.43 ± 0.63 kg, and the pubertal phase goats were about 3 months old, weighing 7.82 ± 0.78 kg. Blood samples of the goats for experimentation were collected for hormone detection 1 day before sacrifice in Taian Central Hospital. After evaluation of the hormone level and selection of pubertal goats in the estrus period, the goats were then sacrificed after anesthesia with 0.1 ml xylazine hydrochloride injection (Muhua, China, lot number 150804). Hypothalamus samples were collected in liquid nitrogen before use. Total RNA was extracted (stored at −80°C) according to the EZNA Tissue RNA Kit instruction manual (Omega-Biotech, Doraville, USA). RNA concentration and purity were measured with a NanoDrop 2000 Spectrophotometer (Thermo Fisher Scientific, Wilmington, DE, USA). RNA integrity was assessed using the RNA Nano 6000 Assay Kit with the Agilent Bioanalyzer 2100 system (Agilent Technologies, CA, USA).

### RNA library construction and RNA sequencing (RNA-Seq)

The cDNA library was constructed following the manufacturer's instructions with an NEB Next Ultra RNA Library Prep Kit for Illumina (NEB, E7530) and NEB Next Multiplex Oligos for Illumina (NEB, E7500). Sequencing was then performed using a paired-end 125-cycle rapid run on the Illumina HiSeq2500 (Illumina Inc., San Diego, CA, USA). Low-quality reads were removed, and the clean reads were filtered from the raw reads and mapped to the caprine genome using Tophat2 software. The transcriptome data have been deposited with the National Center for Biotechnology Information Gene Expression Omnibus (GEO, http://www.ncbi.nlm.nih.gov/geo) under accession number SAMN09205053. Gene expression levels were estimated based on the FPKM (Fragments Per Kilobase Million) values obtained using Cufflinks software. The discrepant genes were analyzed only with an absolute value of log2 (fold-change) ≥ 2 and an FDR (False Discovery Rate) < 0.01.

### DNA preparation for reduced representation bisulfite sequencing (RRBS), quantification and sequencing

The genomic DNAs (3 juvenile phase, 3 puberty phase) were extracted using a TIANamp genomic DNA kit (Tiangen, Beijing, China) following the manufacturer's instructions. The construction of the DNA methylation library was performed at Biomarker (Beijing, China) using RRBS. In detail, DNA from each sample was measured and diluted to a standard concentration. Subsequently, two independent pools were constructed, and each pool contained the same quantity of DNA from the goats in the same phase. Then, the two pooled samples were sonicated to produce 100–500 bp DNA fragments. After DNA underwent end repair, phosphorylation and A-tailing with the Paired-End DNA Sample Prep kit (Illumina, San Diego, CA, USA), it was ligated to Illumina sequencing primer adaptors. The final library was generated by PCR amplification, enriching for fragments with adapters on both ends and RRBS was performed by Illumina HiSeq X Ten (Illumina Inc., San Diego, CA, USA). The clean reads were aligned to the caprine reference genome and produced by BS-seeker2 v.2.0.8 using Bowtie2 v.2.1.0 (12) in local alignment mode and no more than 2 mismatches per read. Methylation status was determined using weighted methylation level (13). The differentially methylated regions (DMRs) were produced, with site coverage depth of more than 10×, at least 3 different methylation sites and a Fisher's exact test value of *P* < 0.05 using MOABS (14). The RRBS sequences were submitted to the Gene Expression Omnibus (GEO) of the National Center for Biotechnology Information under the study accession number SAMN09205053.

### Bioinformatics analysis

DEGs and DMR genes enrichment analysis between juvenile and pubertal Jining Gray goats was carried out using DAVID Bioinformatics Resources (version 6.8, https://david.ncifcrf.gov/home.jsp). FPKM of independent sample and DEG correlation analysis were implemented by Java cluster (15). All the genes that had similar gene expression patterns were classified by STEM software (version 1.3.9, http://www.cs.cmu.edu/~jernst/stem); meanwhile, the correlation coefficients of significant clustered profiles were calculated using the MultiExperiment Viewer (version 4.9.0, http://www.tm4.org). All of the DEGs were analyzed using Cytoscape ClueGO plug-in (version 2.3.2, http://apps.cytoscape.org/apps/cluego) as a complementary analysis method. Only Benjamini-corrected values of *P* < 0.05 were considered statistically significant. DEG protein correlation was predicted by cytoscape network.

### Identification of DEG by real-time PCR

DEG expression discrepancy was then verified by quantitative PCR. The total RNA was extracted using TRIzol reagents (Sigma, St. Louis, USA) according to the manufacturer's instruction book. Subsequently, cDNA was synthesized by using Primescript RT reagent (TaKaRA Bio Inc., Otsu, Japan) in a 20 μL total volume, according to the manufacturer's instruction book. Expression of six genes was quantified using SYBR Premix Ex Taq (TaKaRA Bio Inc., Otsu, Japan) in an Agilent Mx3000P system (Agilent Co., Wilmington, Delaware, USA) in a total volume of 25 μL, containing 12.5 μL 2× Pre Ex Taq, 0.5 μL ROX II, 2 μL cDNA, 0.5 μL each of the forward and reverse primer (10 μM) and dH_2_O. All the primers used in this study are detailed in Table 9.

### Statistical analysis

DEG expression analysis was presented as mean ± SEM and analyzed by one-way ANOVA test. Gene ontology (GO) and KEGG analysis were performed by Fisher's *t*-test. *P* < 0.05 was considered as significantly different.

## Results

### Estrous goats were evaluated by serum gonadal hormone level

Estrous behavior was evaluated by the serum sex hormone level in the Jining Gray goat. Average gonadal hormones were evaluated by entire herd and FSH>20 U/L was identified as a sign of oocyte maturity. Estrous goats were recognized after a comprehensive consideration referring to the LH, estrogen and progesterone levels. Table 1 lists the serum gonadal hormone levels of the goats used in this study.

**Table 1:** Comparison of gonadotropic hormone concentration between juvenile and pubertal Jining Gray goats.

**Sample**	**Estrogen**	**LH**	**FSH**	**PRL**	**Progesterone**	**Testosterone**
Juvenile 01	120.52	13.22	16.97	4.21	1.78	69.29
Juvenile 02	109.83	8.65	5.99	4.79	1.97	63.46
Juvenile 03	46.81	12.26	5.98	3.53	0.63	6.89
Puberty 01	86.91	25.82	37.52	5.09	1.46	127.02
Puberty 02	68.52	29.59	28.17	4.39	1.32	876.4
Puberty 03	63.57	24.48	32.55	5.14	0.63	119.5

*LH, Luteinizing Hormone (U/L); FSH, Follicle-Stimulating Hormone (U/L); PRL, Prolactin (nmol/L); Estrogen, (pmol/L); Testosterone, (nmol/L); Progesterone, (nmol/L)*.

### Landscape of the mRNA transcriptomes of hypothalamus tissue

**Figure 1:**
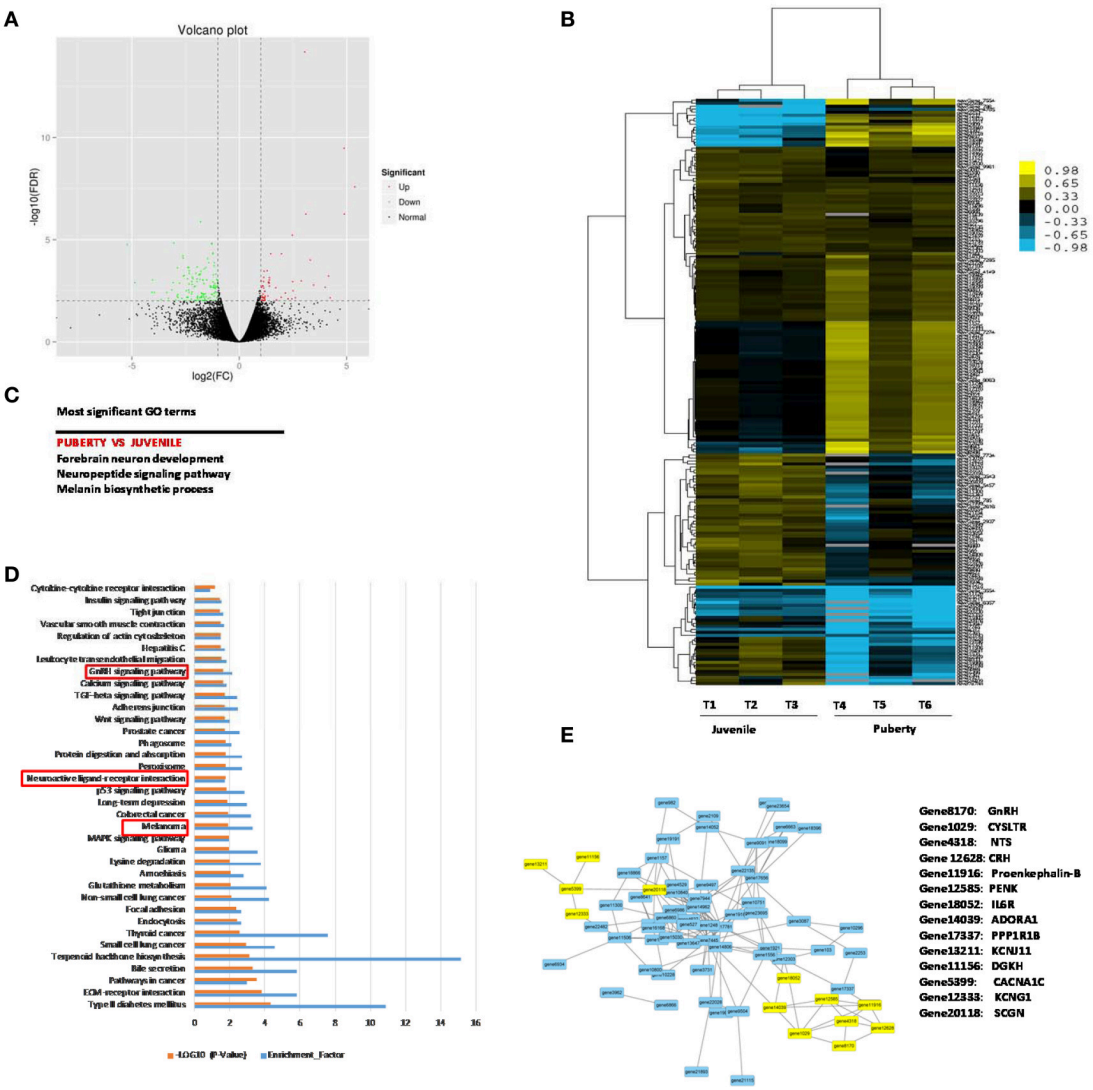
Gene profile analysis of Jining Gray goat hypothalamus between juvenile and pubertal phase. **(A)** Volcano plot of all the genes detected in the experiment. Green spots represent down-regulation, red spots represent up-regulation. **(B)** Hierarchical clustering analysis for DEGs between juvenile and pubertal phase. **(C)** Potential pathways that influence sexual precocity characteristics in Jining Gray goat by GO term analysis. **(D)** KEGG signal pathway enrichment analysis of DEGs. [−log (Q-value)>1.3 were recognized as significantly different]. **(E)** Potential correlation analysis of DEG protein relationship.

Two groups of Jining Gray goats were sacrificed and their hypothalami were used for sequencing to identify genes that might influence sexual precocity performance. High-throughput RNA-Seq generated 21.7 Gb data for the two phases of Jining Gray goats, and more than 77.71% reads were successfully aligned to the goat genome. All samples had at least 91.04% reads equal to or exceeding Q30 (Table 2). A volcano plot was created to characterize gene expression levels, red dots represent up-regulated genes and green dots represent down-regulated genes (Figure 1A). DEGs were then screened according to the criterion of fold-change ≥ 2 and FDR ≤ 0.01, and hierarchical clustered maps revealed 180 annotated genes that are differently expressed in these two phases (Supplementary Table 1, Figure 1B). GO terms associated with these genes revealed their specialized roles in forebrain neuron development, the neuropeptide signaling pathway and the melanin biosynthetic process (Figure 1C, Table 3). Subsequently, KEGG enrichment of DEGs was evaluated and potential pathways including the melanoma pathway, the GnRH signal pathway and the neuroactive ligand-receptor interaction pathway (*P* < 0.05) were identified (Figure 1D). The relationships of the proteins encoded by these DEGs were then assessed to explain the regulatory network (Figure 1E, Table 4, the yellow spots represent the pathway referred to in Figure 1D).

**Table 2:** Summary of RNA-Seq metrics from transcriptomes between juvenile and pubertal Jining Gray goats' hypothalami.

**Sample**	**Total reads**	**Mapped reads**	**Mapped ratio (%)**	**Uniq mapped reads**	**Uniq mapped ratio (%)**	**GC content (%)**	**%≥Q30**
Juvenile 01	34,489,232	27,224,698	78.94	26,030,113	75.47	50.29	91.54
Juvenile 02	42,172,368	33,940,726	80.48	32,627,715	77.37	50.13	91.81
Juvenile 03	35,755,664	27,784,233	77.71	26,499,447	74.11	49.77	91.04
Puberty 01	34,280,210	26,761,566	78.07	25,443,511	74.22	50.22	91.49
Puberty 02	32,118,330	25,114,652	78.19	23,975,747	74.65%	50.47%	91.65
Puberty 03	39,187,148	30,965,637	79.02	29,501,769	75.28%	49.90%	91.77

**Table 3:** DEGs involved in signal pathways analyzed by GO terms.

**GO terms**	**Term number**	***P*-Value**	**Genes**
Forebrain neuron development	GO:0021884	3.81E-06	RAPGEF2, GBX2, LHX6
Neuropeptide signaling pathway	GO:0007218	0.000566	GPR124, BAIAP2, PENKB, CALCA, PENK
Melanin biosynthetic process	GO:0042438	0.002639	DDT, CITED1

**Table 4:** The genes involved in gonadal hormone secretion.

**KEGG pathway**	**GeneID**	**Annotation**	**FDR**	**log2FC**	**Regulated**
Neuroactive ligand-receptor interaction	gene4318	*NTS*	4.90E-05	1.464	Up
	gene14039	*ADORA1*	2.14E-03	−1.23	Down
	gene12628	*CRH*	1.05E-03	2.884	Up
	gene11813	*UCN3*	2.60E-08	5.376	Up
GnRH signaling pathway	gene8170	*GNRH1*	3.36E-10	4.875	Up
	gene5399	*CACNA1C*	5.93E-03	−1.169	Down
Melanoma	gene1921	*E2F2*	8.15E-04	−2.16	Down
	gene7445	*PDGFRB*	9.39E-04	−1.707	Down

### Changes in genomic methylation patterns of hypothalamus tissue

**Figure 2:**
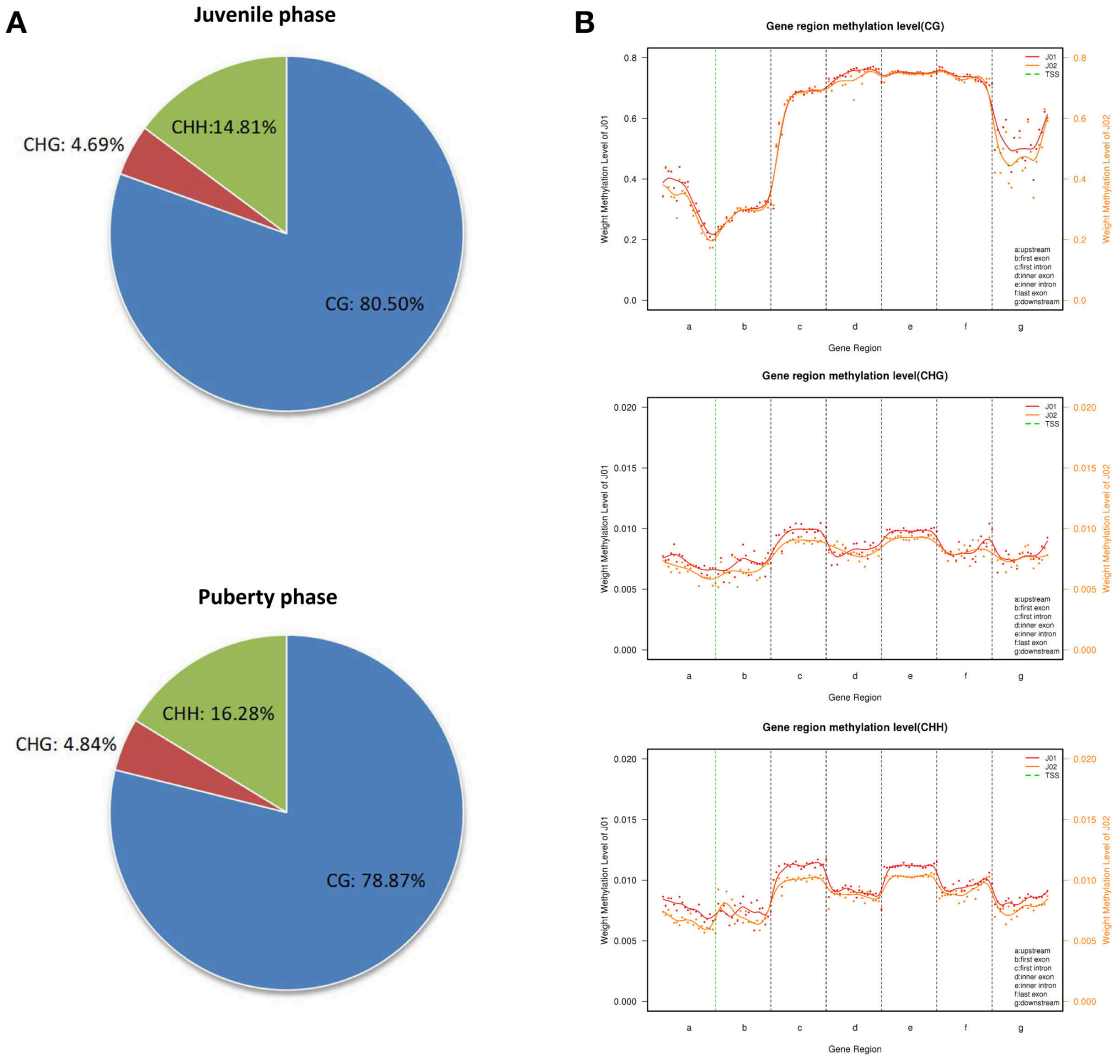
Genome methylation comparison between juvenile and pubertal phase Jining Gray goats. **(A)** Methylation pattern analysis between juvenile and pubertal goats. **(B)** Methylation distribution analysis between juvenile and pubertal goats.

To further understand the role of DNA methylation in regulating the transcription of genes related to the sexual precocity of Jining Gray goats, genomic DNA RRBS on the mixed pools of the two phases was carried out. More than 3.4 GB clean data were obtained in each group, with 49.66 and 54.98% clean reads uniquely mapped. An over 99.42% conversion ratio of these two mapped reads was found and all samples had at least 85.25% reads equal to or exceeding Q30 (Table 5). CG methylation is the dominant pattern relative to the CHG and CHH patterns in both juvenile and pubertal phases, accounting for 80.50 and 78.87%, respectively (Figure 2A). The genome-wide CG methylated pattern in genes was obviously different from that of CHG and CHH, with low methylated regions mainly existing in the upstream of TSS(Transcription Start Site) (Figure 2B). Additionally, comparison of methylated regions (CG/CHG/CHH) showed that for the puberty phase, methylation was lower than for the juvenile phase both in upstream and downstream regions. MeDIP-Seq (methylated DNA immunoprecipitation sequencing) reads were then detected in most caprine chromosomal regions, and CG patterns covered over 56.90% in the hypothalamus of both juvenile and pubertal Jining Gray goats (Table 6).

**Table 5:** Summary of RNA-Seq methylation information between juvenile and pubertal Jining Gray goats' hypothalami.

**Sample**	**Clean reads**	**Mapped reads**	**Mapped ratio (%)**	**Conversion rate (%)**	**GC %**	**%≥Q20**	**%≥Q30**
Juvenile	35,004,578	17,382,147	49.66	99.42	29.62	90.99	85.25
Puberty	34,884,715	19,179,092	54.98	99.45	28.37	92.46	86.45

**Table 6:** Summary of methylation coverage in genomes in the hypothalami of juvenile and pubertal Jining Gray goats.

**Sample**	**mCHG**	**mCHH**	**mCpG**	**mC-total**	**mCHG (%)**	**mCHH (%)**	**mCpG (%)**
Juvenile	2,846,152	6,991,558	73,604,599	83,442,309	1.00	1.10	57.70
Puberty	2,692,629	7,082,870	67,860,606	77,636,105	1.00	1.00	56.90

### Enrichment in genes with a DMR

**Figure 3:**
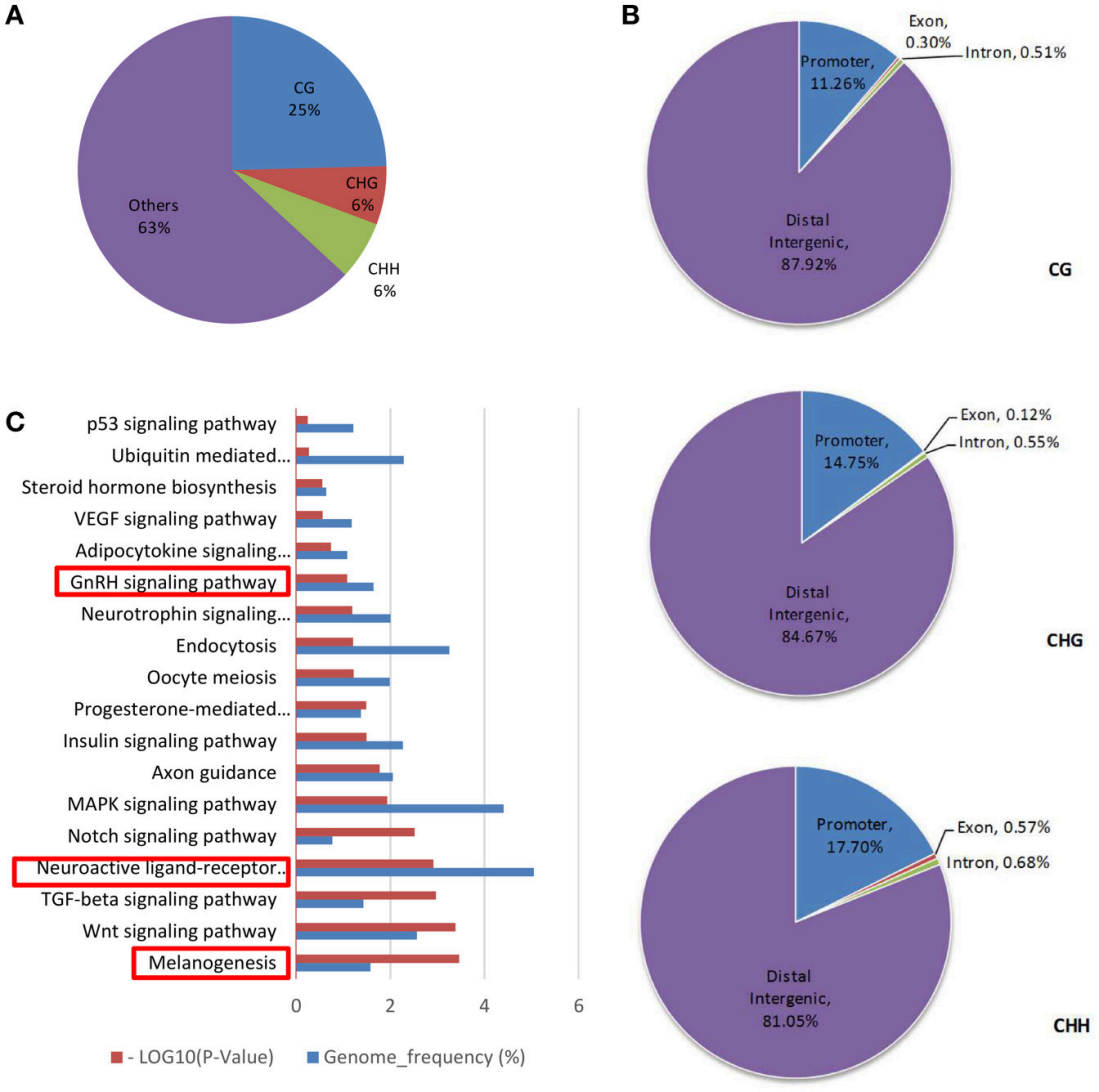
DMR gene methylation analysis. **(A)** DMR gene methylation pattern analysis. **(B)** Analyzed DMR genes methylated site distribution in CG/CHG/CHH patterns. **(C)** KEGG analysis of DMR genes in the CG pattern [−log(Q-value)>1.3 was recognized as significantly different].

Genes with a DMR were obtained after enriching for DMRs in the genomic DNA. CG, CHG and CHH methylated patterns occupied 25, 6, and 6% in DMR genes, respectively (Figure 3A). Then, the methylation distribution of DMR genes was analyzed and the data were indicative that more than 80% methylation exists in the distal intergenic region, 10% methylation in the gene promoter and less than 1% in exon regions, no matter whether in CG, CHG, or CHH methylated patterns (Figure 3B). GO term analysis on DMR genes (Supplementary Table 2) identified two key signals: GnRH and neuroactive ligand receptor interaction pathways that regulate estrous and oocyte development (*P* < 0.05), and the melanogenesis pathway that influences estrous types (Figure 3C, Table 3).

### Correlation analysis between DEGs with DMR genes

**Figure 4:**
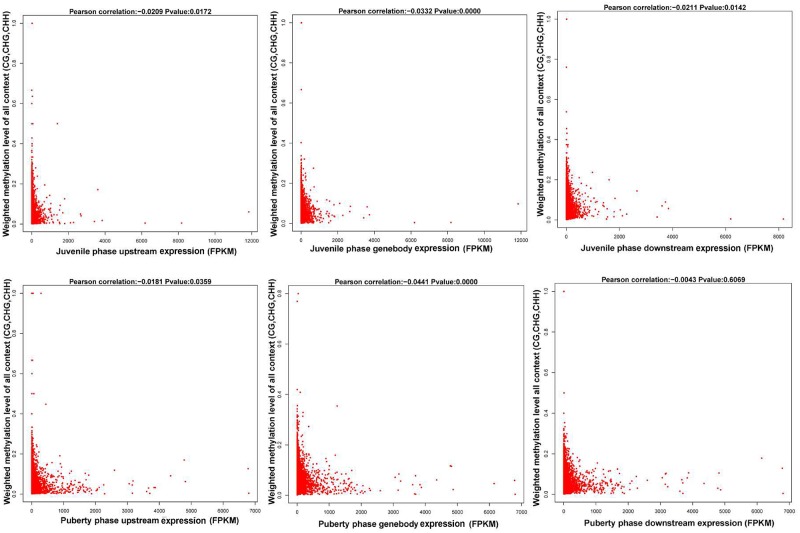
Correlation analysis between gene expression and its methylation distribution.The first row is the juvenile phase and the second row is the puberty phase of Jining Gray goats. The left column of this figure is the methylation level of the gene upstream region, the medial one is the gene body region and the right one is downstream of the gene. All the data are indicative that the methylation level of DMR genes correlates negatively with their expression levels (Pearson's *r* < 0, *P* < 0.05 for juvenile; and Pearson's *r* < 0, *P* < 0.05 for puberty).

**Figure 5:**
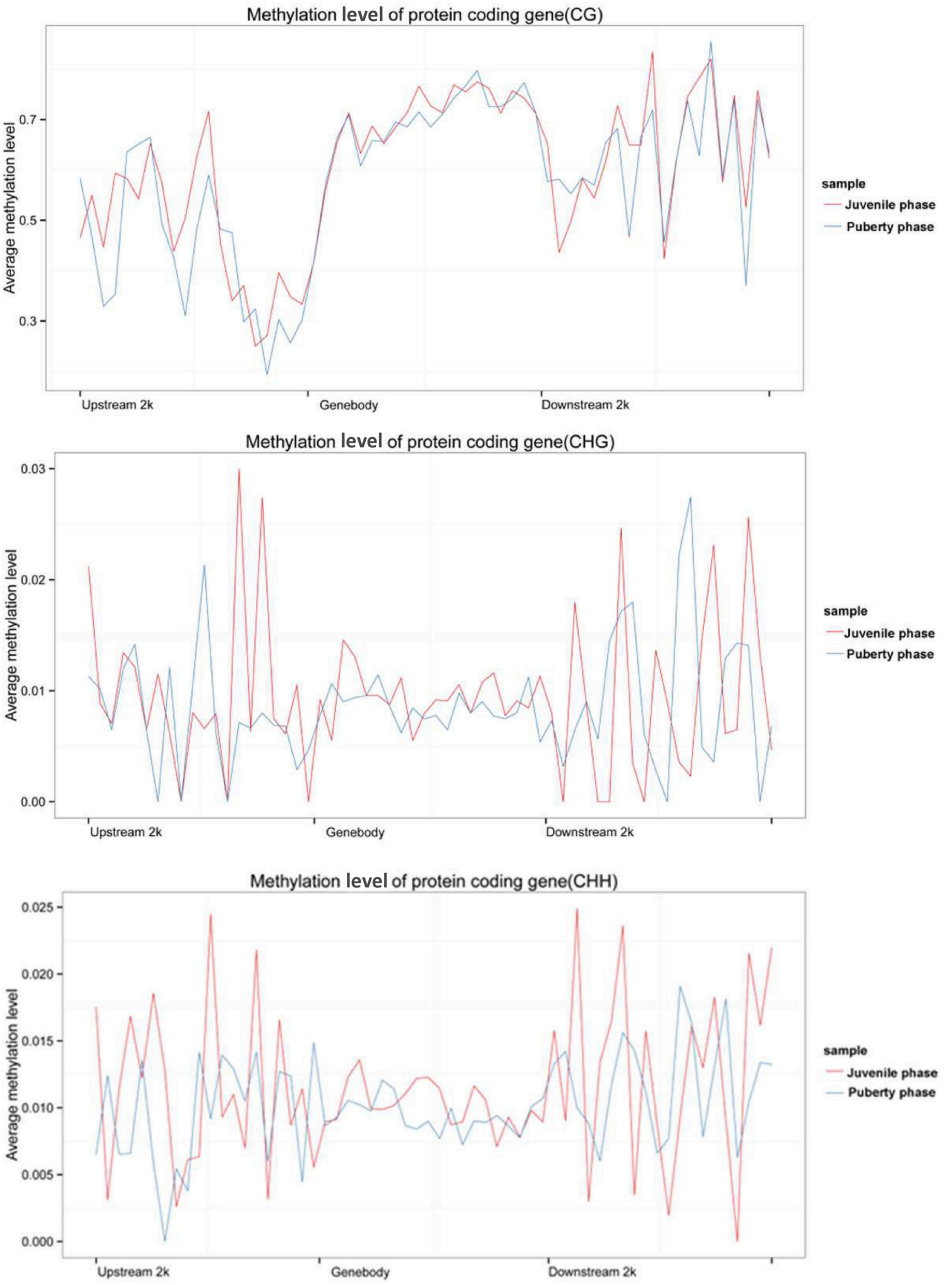
Analyzed DEGs average methylation levels in the upstream, gene body, and downstream regions of the genes. Upper image is the CG methylated pattern, the gene body region shows a higher level of DNA methylation than the 5′and 3′ flanking regions of genes. The DNA methylation level decreased dramatically proximal to the TSS, increased sharply toward the gene body region and plateaued until the downstream. Additionally, its level in puberty phase is lower than that in juvenile phase. The medium and bottom images are CHG and CHH patterns, respectively.

**Figure 6:**
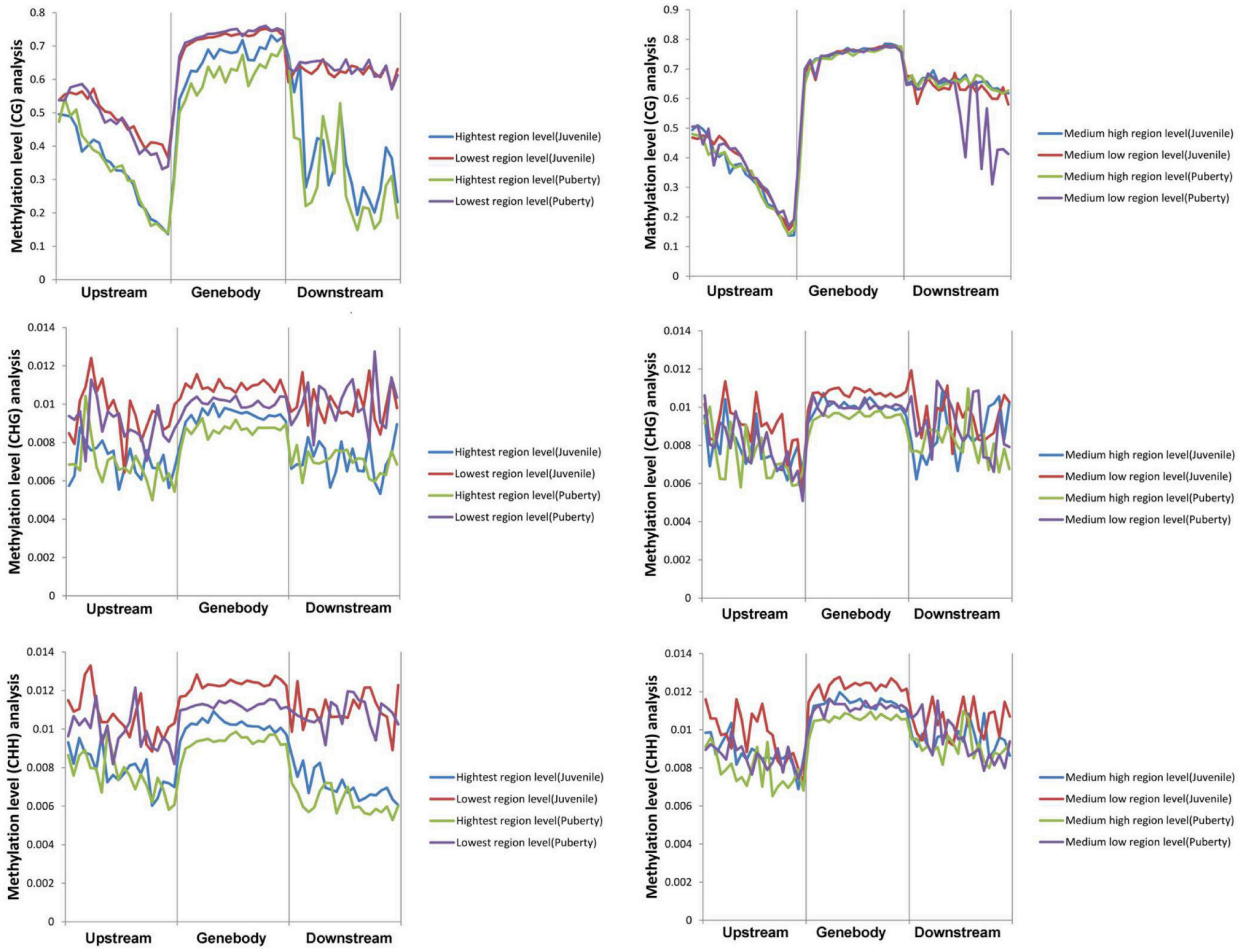
Correlation analysis between DEGs expression and its methylation changes in CG/CHG/CHH patterns. Correlation analysis of DEG expression level with its methylation level in the CG pattern (upper image). Correlation analysis of DEG expression level with its methylation level in the CHG pattern (middle image). Correlation analysis of DEG expression level with its methylation level in the CHH pattern (bottom image). The left column is highest and lowest expressed DEGs, and the right one is medium high and medium low expressed DEGs.

We calculated each gene's methylation level and expression level in the hypothalamus of juvenile and pubertal Jining Gray goats, and determined their distribution characteristics in terms of DNA methylation and mRNA transcriptome. The data were indicative that the methylation level of DMR genes correlates negatively with expression level (Pearson's *r* < 0, *P* < 0.05 for juvenile; and Pearson's *r* < 0, *P* < 0.05 for puberty) (Figure 4). Subsequently, average methylation levels of DEGs were analyzed in the upstream, gene body and downstream regions. For the CG methylated pattern, the gene body region shows a higher level of DNA methylation than the 5′ and 3′ flanking regions of genes. The region around the TSS is crucial for the regulation of gene expression. The DNA methylation level decreased dramatically proximal to the TSS, increased sharply toward the gene body regions and plateaued until the downstream. Additionally, its level in puberty phase is lower than that in juvenile phase (Figure 5, upper). For the CHG and CHH patterns, no obvious changes were found in these three regions (Figure 5, medium and bottom). We divided the genes equally into four groups (lowest, highest, medium high, medium low) according to their expression levels and counted the genes of each group in each sample. The DNA methylation profiles in and around gene bodies were compared among these four gene expression levels. A clearly negative and monotonic correlation was found between DNA methylation levels around the TSS of genes and gene expression levels. For CG or non-CG patterns, the methylation level of highly expressed genes was obviously lower than that that of low expressed ones, especially in their TSSs. Conversely, the intermediate expressed genes have little differences in their methylation no matter whether they are medium high or low expression genes (Figure 6).

### Integrated analysis of DEGs and DMR genes in different patterns

**Figure 7:**
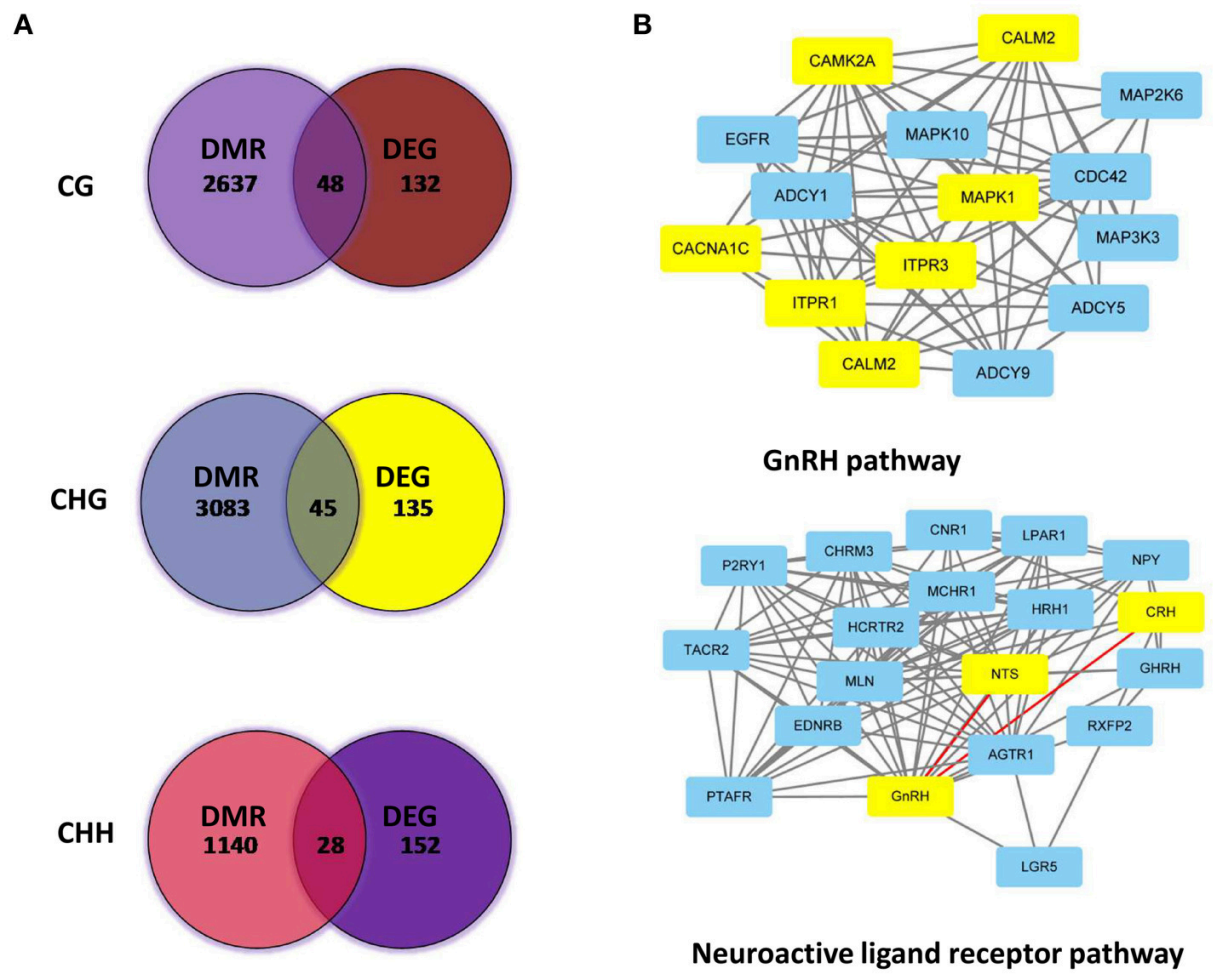
Associated analysis of DEGs and DMR genes in different patterns. **(A)** Analyzed Venn diagrams between DEGs and DMR genes in different methylation patterns. **(B)** Integrated analysis of DEGs and DMR genes that are involved in GnRH and neuroactive ligand receptor pathways.

DMR genes and DEGs were then subjected to integrated analysis by Venn diagrams. Figure 7A shows DEGs for different methylation patterns in the hypothalamus. The upper figure indicated 48 DEGs were involved in the CG methylated pattern (Supplementary Table 3), while 45 genes (Supplementary Table 4) were involved in the CHG patterns (middle) and 28 genes (Supplementary Table 5) in CHH methylated patterns (bottom). Subsequently, DEGs and DMR genes that are involved into GnRH and neuroactive ligand receptor pathways were analyzed and their interactions were revealed (Figure 7B, the yellow-labeled genes are the DEGs; Supplementary Table 6).

### Genes related to sexual precocity and affected by DNA methylation

**Figure 8:**
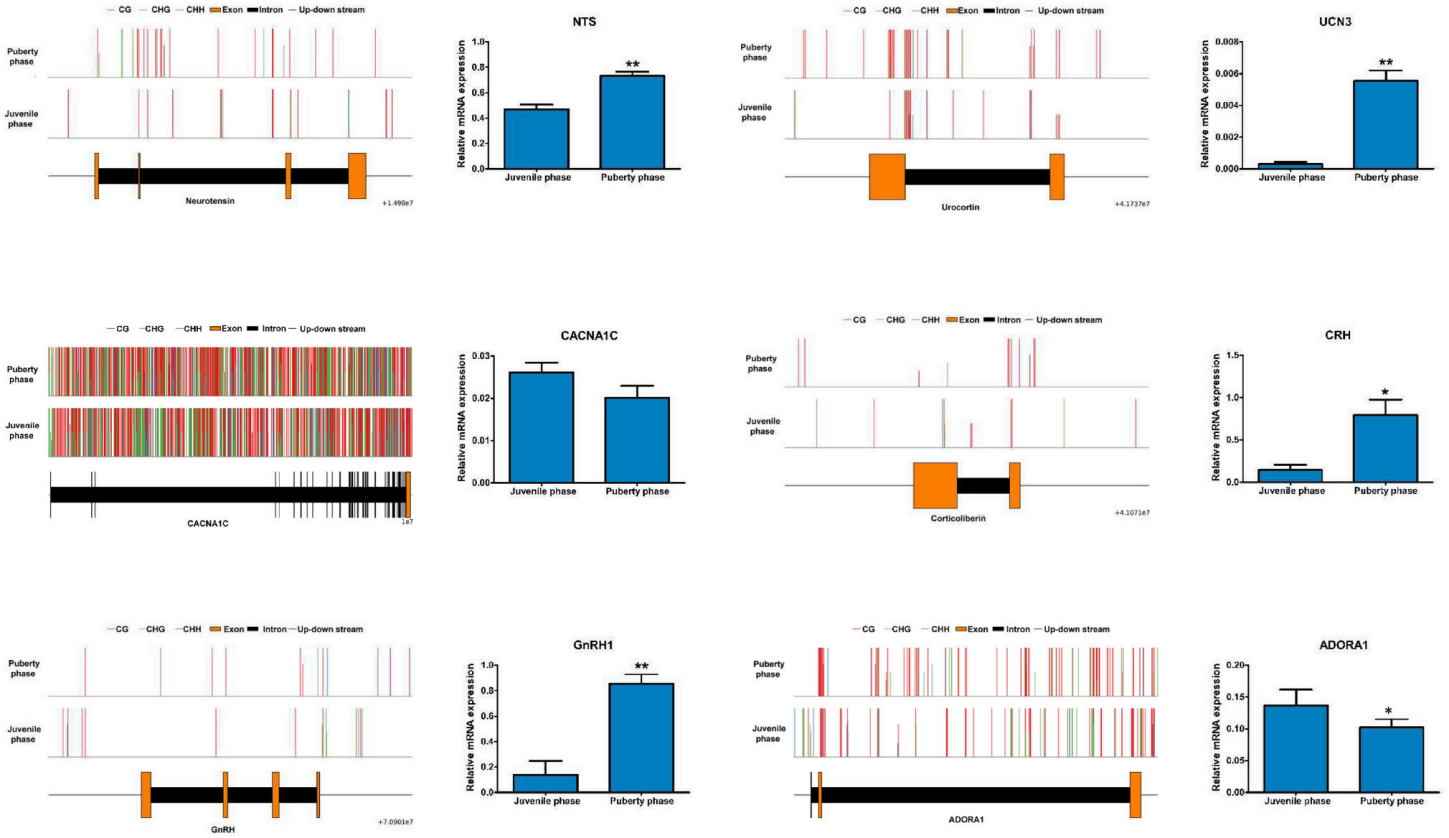
Identification of key genes and their methylation levels that are involved into gonadal hormone secretion. The expression of *NTS, UCN3, CRH*, and *GNRH1* in the hypothalamus was significantly higher in puberty than that in the juvenile phase of Jining Gray goats. Conversely, the expression of *CACNA1C* and *ADORA1* was significantly lower in puberty than that in the juvenile phase of Jining Gray goats. The methylation maps of NTS, UCN3, GnRH1, CRH, and ADORA1-encoding genes exhibited few methylated sites in the promoter regions, while *CACNA1C* genes exhibited abundant methylated sites. DEG gene expression tests were replicated three times, the data were presented as mean ± SEM and were analyzed by one-way ANOVA test (*P < 0.05, **P < 0.01).

Among the genes involved in regulating sexual hormone secretion, the expression of *NTS, UCN3, CRH* and *GNRH1* in the hypothalamus was significantly higher in puberty than that in the juvenile phase of Jining Gray goats. Conversely, the expression of *CACNA1C* and *ADORA1* was significantly lower in puberty than that in juvenile phase of Jining Gray goats, which is identical to the data from transcriptome analysis (Figure 8). Compared with the abundant methylated sites exhibited in *CACNA1C* genes, methylation maps of *NTS, UCN3, GNRH1, CRH*, and *ADORA1* genes exhibited few methylated sites in their promoter regions (Promoter 0-2KB), which is in accordance with CG methylated patterns of DMR genes (Figure 8, Table 7).

**Table 7:** Genes involved in gonadal hormone secretion.

**Gene ID**	**Annotation**	**Regulated**	**FDR**	**log2FC**	**Meth_stat**	**Meth site**
gene5399	*CACNA1C*	down	5.93E-03	−1.17	up	Promoter (1-2kb)
gene12628	*CRH*	up	1.05E-03	2.88	up	Distal Intergenic

**Figure 9:**
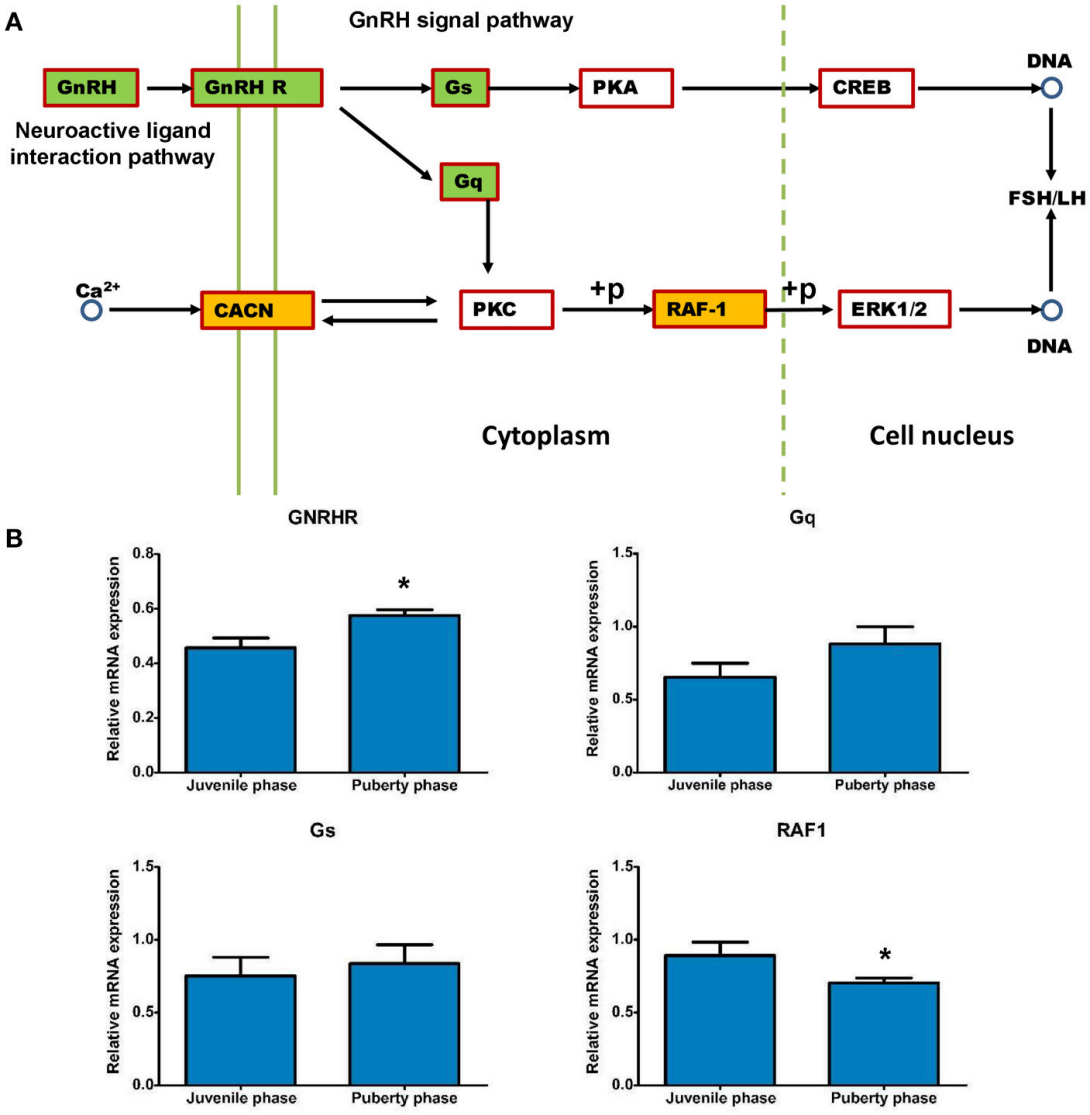
Relevant genes methylated and their expression changes that are involved in the GnRH signal pathway. **(A)** The potential GnRH signal pathway changes that are caused by DMRs and DEGs. **(B)** Gene expression analysis of methylated *Gs* and *Gq* genes that influence the GnRH pathway. DEG gene expression tests were replicated three times, the data were presented as mean ± SEM and were analyzed by one-way ANOVA test (*P < 0.05).

The expression differences that are involved into GnRH and neuroactive signals pathways were analyzed and are shown in Figure 9A. Expression of *GNRH, Gs, Gq* and *GNRHR* genes was increased, while that of *CACNA1C* and *RAF-1* genes was decreased in puberty compared to the juvenile phase (Figures 8, 9B). Then, methylation changes of all these genes were analyzed and only for Gs and Gq genes were methylation changes found, which may cause expression to be slightly increased (Table 8).

**Table 8:** Methylation distribution of the key genes that influence FSH and LH secretion.

**Gene name**	**Gene Id**	**Meth direction**	**Meth site**	**Gene start**	**Gene end**	**Gene length**	**Distance to TSS**
Gs	gene11987	DOWN	Distal Intergenic	55500135	55505188	5054	52378
Gq	gene3636	DOWN	Distal Intergenic	38587325	38692168	104844	−111528

**Table 9:** The primers used in this study.

**Primer name**	**Sequence (5′–3′)**	**Length**	**Annealing temperature**
CITED-F	TGGATGAAGAGGTGCTGA	121	56
CITED-R	TGCCGTGAAGTCAAACTC		
UCN3-F2	ATACCTACACACACCAGCG	144	60
UCN3-R2	ATGACCTTTGCCTCTCCT		
CRH-F	TTCCACCTCCTCCGAGAAGT	135	56
CRH-R	TTTAGCCAAACGCACCGT		
ADORA1-F1	ATCACTGGCTGCTGGATT	202	56
ADORA1-R1	CCCACACGAAGAAGTTGAA		
NTS-F	AGTAAGGCAAGTGTTCCC	250	56
NTS-R	TCCTGAATCAACTCCCAG		
CACNA1C-F	TAGAGCAAGCGACCAAAG	239	56
CACNA1C-R	CGATGATGGCGTAGATGA		
GNRH1-F	TGCTGACTTTCTGTGTGG	159	56
GNRH1-R	GCTTAGGTTCTACTGGCTG		
GnRHR-F	TCAACAGCAGCATCCTAC	237	54
GnRHR-R	GCATAACAATCAGAGTCTCC		
RAF1-F	GCTTACGGACCCTTCTAA	200	54
RAF1-R	AGGCAGCATCAGTATTCC		
Gq-F	GGAATGCTATGACAGACG	113	54
Gq-R	GCACATCTTGTTGCGTAG		
Gs-F	CCATCATCTTCGTGGTTG	101	54
Gs-R	CAGCCATCTGTTGTTCCA		
GAPDH-F1	AGCCGTAACTTCTGTGCT	245	51–58
GAPDH-R1	TTCTCTGCCTTGACTGTG		

## Discussion

To explore genes and pathways affecting the sexual precocity of the reproductive performance of Jining Gray goats, hypothalamus transcriptomes was evaluated in this study. The whole genome RNA-Seq obtained 180 DEGs that were mainly enriched in the GnRH and neuroactive ligand interactive signal pathways. Subsequent RRBS identified 79 genes (DMRs) involved in these two pathways. Integrated analysis of genome methylation and transcriptome revealed that the methylation levels of DMR genes correlated negatively with their expression levels. Integrated analysis of DMRs and DEGs that are involved in GnRH and neuroactive ligand signal pathways identified an integrated regulation network. We then verified some key genes that affect the known signals, differences in the expression of these genes was suggestive that changes in DNA methylation influence gene expression, which ultimately leads to the sexual precocity of Jining Gray goats.

Mammalian body temperature detection has been recognized as a common method for estrus determination, especially in large-scale animal farming (16). However, the accuracy is lower relative to gonadal hormone detection. A previous study reported the concentration of sexual hormones was a key characteristic for mammalian sexual maturity and their secretion concentrations were changed during the estrous period (17). In this study, the goats that secreted sufficient FSH were identified as pubertal goats and used for experiments, which is the same as for the previous report.

Hypothalamus transcriptome data were analyzed, and eight critical genes and three potential signal pathways were obtained. Among these genes, *E2F2* and *PDGFRB* were widely recognized as key genes that impact melanoma secretion (18, 19). GnRH1 was mainly secreted from the hypothalamus and was known to play some roles in the pituitary, especially regulating FSH and LH secretion (20). *CACN1C* genes coordinatively participate in ERK activation and cause the increase of FSH and LH secretion in the GnRH signal pathway (21). *NTS, ADORA1, CRH*, and *UCN3* genes mainly contribute to the neuroactive ligand-receptor interaction pathway, as previously reported. GO terms and KEGG analysis was suggestive that the signals that cause the sexual precocity of Jining Gray goats lie in the melanoma pathway, neuroactive ligand-receptor interaction pathway and GnRH pathway, which also has been reported in previous studies (22). It has been well studied that DNA methylation plays an important role in the regulation of gene expression (23). In this study, genomic methylation was investigated to find the DMR genes and the related pathways. Methylation sequencing depth covered the whole genome and was adequate for further study, as previously reported (11). Meanwhile, methylation patterns exhibited CG methylation as the main pattern in mammals and its distribution is mainly on the promoter and 3-UTR regions of DEGs, which is consistent with another study (11, 23). Subsequently, distributions of DNA methylation in DMR genes were analyzed and the methylation mainly occurred in intergenic and promoter regions, which was indicative of a negative correlation between methylated effects and gene expression, as reported (24, 25). DMR gene enrichment identified three key signal pathways, which is consistent with DEG enrichment in this study. DMR genes enriched in GnRH and neuroactive ligand-interaction pathways were analyzed, 80 genes were obtained and their potential relationships were predicted. These genes may play important roles in the pathways that may be associated with FSH and LH secretion and the onset of puberty, although further studies are necessary to elucidate the functional roles of these genes during puberty (4).

Generally, DEGs with high expression levels are often associated with a relatively lower promoter methylation, especially prior to TSS regions. The correlation between gene expression and methylation levels of the gene body remain unclear (26). Previous studies assumed that DNA methylation in the gene body is positively correlated with gene expression level, because DNA methylation might alter chromatin structure and transcription elongation efficiency (27, 28). In this study, DEGs and their methylation levels were evaluated to confirm their correlation. Relatively lower promoter and higher gene body methylation levels confirmed the correlation, as a previous study reported (28).

The methylation level may alter gene expression, which may affect the entire pathway and finally lead to physiological process changes. In this study, DMR genes and DEGs that are involved in GnRH and neuroactive ligand interaction pathways were analyzed to find the entire pathway changes that were associated with FSH and LH secretion and pubertal onset. The analyzed data were indicative that differentially methylated genes almost covered all the pathways related to FSH and LH secretion. The key genes that are involved in these pathways were evaluated by their expression and methylation levels, and the data exhibited that lower promoter methylation causes high gene expression, as previously reported (26, 29). Subsequently, integrated analysis on the key genes *Gs, Gq, RAF-1*, and *GNRHR* by expression and methylation distribution was indicative that the distal intergenic methylation may influence the gene expression, which is the same as previously reported (28). However, the mechanisms need further exploration.

Previous study reported that DNA methylated level of hypothalamus was evaluated by Yang's work in Anhui goat in its pubertal phase (4). There are some obviously differences between their work with us. For one thing, Yang's paper used Anhui goats while in this assays we used Jining Gray goats. Anhui goats reach puberty (time to first ovulation) at the age of 5 months while Jining Gray goats reach puberty at the age of 2 months. For another, Yang' paper mainly focus on the methylated changes between pubertal and Juvenile goats, so in his study he screened DMRs genes that may influence pubertal onset. Based on the specific DMRs, two genes (*NLRC5, PLCXD3*) were found to be hypermethylated and five genes (*PPM1D, CD226, SMOC1, GRID1, and LOC10219031*) became hypomethylated during the onset of puberty. He also found methylated level changes in *DHRS3, NLRC5, CIB4, DOCK6*, and SCO-spondin during prepubertal and pubertal stages (4). Our study mainly focused on the transcriptomes and their relationship with DNA methylomes in the hypothalamus that related to sexual precocity in Jining Gray Goats. Different genes were screened by different analytical method.

In summary, the transcriptome and methylome landscape in the hypothalamus reveals key genes and pathways that influence sexual precocity in Jining Gray goats. Expression of DEGs revealed and confirmed *NTS, ADORA1, CRH, UCN3, GNRH1*, and *CACNA1C* were the potential genes that impact FSH and LH secretion. Analysis of DMR genes revealed the potential regulation network that influences pubertal onset. Correlation analysis verified DMR gene methylation levels correlate negatively with expression levels. Associated analysis between transcriptomes and methylomes identified genes and pathways that influence FSH and LH secretion and pubertal onset. This study provides some candidates in the identification of the key genes underlying sexual precocity in Jining Gray goats.

## Author contributions

FS, GC, and YJ designed and drafted the manuscript. XG and YaW carried out animal care, prepared samples and performed the experiments. YuW and FS performed the data processing and biological information analysis. FS, GC, and YJ conceived the study and the experimental design and helped draft the manuscript. All authors read and approved the final manuscript.

### Conflict of interest statement

The authors declare that the research was conducted in the absence of any commercial or financial relationships that could be construed as a potential conflict of interest.

## Supplementary material

Supplementary Table 1 DEGs of pubertal Jining Gray goats.

Supplementary Table 2 DMR genes analysis (CG pattern) in the hypothalamus of pubertal Jining Gray goats.

Supplementary Table 3 Integrated analysis of changed genes between DMRs and DEGs in CG patterns.

Supplementary Table 4 Integrated analysis of changed genes between DMRs and DEGs in CHG patterns.

Supplementary Table 5 Integrated analysis of changed genes between DMRs and DEGs in CHH patterns.

Supplementary Table 6 DMR genes that are involved in GnRH and neuroactive ligand signal pathways.

## Abbreviations

DEG: Differentially Expressed Gene
DMR: Differentially Methylated Region
FDR: False Discovery Rate
FSH: Follicle-Stimulating Hormone
GnRH: Gonadotropin-Releasing Hormone
GO: Gene Ontology
LH: Luteinizing Hormone
RNA-Seq: RNA-Sequencing
RRBS: Reduced Representation Bisulfite Sequencing
TSS: Transcription Start Site.
